# Impact of proximal cytoplasmic droplets on quality traits and *in-vitro *embryo production efficiency of cryopreserved bull spermatozoa

**DOI:** 10.1186/1751-0147-54-1

**Published:** 2012-01-12

**Authors:** Janaina T Carreira, Gisele Z Mingoti, Lucia H Rodrigues, Carlos Silva, Silvia HV Perri, Marion B Koivisto

**Affiliations:** 1 Univ Estadual Paulista (UNESP), FMVA, Faculty of Veterinary Medicine, Department of Clinics, Surgery and Animal Reproduction, Araçatuba, Brazil; 2 Univ Estadual Paulista (UNESP), Department of Support, Production and Animal Health, Araçatuba, Brazil; 3 CRVLagoa, Sertãozinho, Brazil

**Keywords:** sperm morphology, proximal cytoplasmic droplets, IVF, fluorescent probes

## Abstract

**Background:**

Proximal cytoplasmic droplets (PCDs), a remnant of germ cell cytoplasm, are common non-specific morphological defects in bovine semen. This study evaluated the effect of higher percentages of PCDs on the quality of frozen-thawed bovine semen, embryo production and early embryo development.

**Methods:**

Three ejaculates from each of five (group 1: PCD ≤ 1%, control) and eight adult *Bos indicus *bulls (group 2: PCD ≥ 24%) were analysed. Semen samples were examined for: post-thaw motility, vigour of movement, concentration, sperm morphology, slow thermoresistance test (STT), membrane integrity, acrosome status, mitochondrial function using fluorescent probes association (FITC-PSA, PI and JC-1) and sperm chromatin integrity using acridine orange assay. Two bulls from group 2, with 28.5% and 48.5% PCD, respectively, and three bulls from the control group, each with 0% PCD, were selected for IVF *(in vitro *fertilisation).

**Results:**

Semen analyses revealed a significant correlation (P < 0.01) between increased rates of PCD and sperm quality traits. Nevertheless, no differences were observed in sperm motility and vigour either before or after the STT or in the percentage of intact acrosomes (analysed by differential interference contrast microscopy (DIC) after STT), but membrane integrity, acrosome status (evaluated with FITC-PSA staining method after thawing) and mitochondrial function were reduced, when compared with group 1 (P < 0.05). The higher incidence of PCD was positively correlated to chromatin damage, especially after three hours of incubation at 37°C. IVF showed similar results for bull C2 (group 1, control) and bull P2 (group 2, group with higher PCDs).

**Conclusion:**

Higher PCD levels influenced spermatozoa quality traits. IVF and embryo development data showed that cleavage, blastocyst formation and blastocyst hatching may have been influenced by the interaction of morphology traits and individual bull effects.

## Background

In the last decade, major advances have been made in the understanding of the biochemical and molecular mechanisms that determine the production of functionally competent spermatozoa [1]. Disturbed sperm-oocyte interaction is the principal cause of low IVF rates in humans and seems to be more associated with sperm defects than with oocyte defects because sperm can penetrate the zona pellucida of oocytes at any stage of maturity and quality. Thus, the proportion of zona pellucida-bound spermatozoa with normal or abnormal morphology is strongly related to IVF success rates [2].

Proximal cytoplasmic droplets (PCDs) can be identified as a regularly shaped remnants of cytoplasm under the plasma membrane in the neck region of the spermatozoa [3]. In a recent study on spermatozoal quality at spermiation in humans, two different kinds of proximal cytoplasmic droplets were described: proximal droplets that reflect epididymal and accessory sex gland alteration and spermatozoa with notable residual cytoplasm (not a cytoplasmatic droplet), indicating an immature cell with improper or incomplete spermiogenesis [4,5]. High percentages of PCDs can be found in the semen of prepubertal bulls, while in mature bulls, high levels of sperm with PCDs are considered a sign of abnormality in spermiogenesis or epididymal sperm maturation [3,6,7].

To have fertilising ability, spermatozoa must acquire a combination of features, such as intact plasma membranes, acrosome integrity, and high mitochondrial membrane potential for the production of adenosine triphosphate (ATP)-generating energy for flagellar beat and motility [8]. Chromatin integrity is another important factor because DNA fragmentation impairs fertility [9]. Combining the results of various sperm function tests improves the reliability of fertility estimation. Current research therefore focuses on identifying a range of tests to assess as many meaningful sperm attributes as possible and doing so easily and at a reasonable cost. Moreover, staining protocols are available for an increasing range of sperm characteristics, including viability, capacitation and acrosome status, mitochondrial activity and chromatin integrity [10]. The objective of this study was to investigate whether a higher percentage of spermatozoa with proximal cytoplasmic droplets could affect the quality and fertilising ability of frozen-thawed semen of *Bos indicus *(Nellore breed) bulls, using routine laboratory sperm assays, plasma membrane, acrosome and mitochondrial status, chromatin integrity and IVF.

## Methods

### Animal selection

A total of 13 mature *Bos indicus *bulls were selected from an artificial insemination centre located in the southeast of Brazil (21°04'52"S, 48°02'24''W); five of the bulls presented normal sperm patterns, with ≤ 1% spermatozoa with proximal cytoplasmic droplets (PCDs) and ≤ 14% total sperm defects (group 1, control, range 4 to 13 years), and eight bulls showed increased levels of PCDs (≥24%) and of total defects (≤48%) (group 2, range 5 to 15 years). Three ejaculates were collected from each bull using an artificial vagina and were cryopreserved (TRIS-egg yolk extender, 7% glycerol in 250 μl French mini-straws) for further evaluation, within a regular twice-a-week collection schedule. The bulls were kept on their native pasture (*Cynodon plectostachyus*) and were given dietary supplementation to optimise their energy balance.

### Semen evaluation

#### Sperm motility, vigour and concentration

Immediately after thawing (35°C for 20 sec), the percentage of progressively motile sperm and their vigour (status of sperm motility), scored on a scale from 0 (without movement) to 5 (fast progressive movement), were determined by subjective estimation using phase contrast microscopy (200×).

Furthermore, sperm concentration (×10^6^) was determined under phase-contrast optics (200×) using a haemocytometer Neubauer chamber.

#### Morphologic abnormalities

Sperm alterations were classified, according to Blom [11] and Barth and Oko [12], into major defects (acrosome defects, proximal droplets, abnormal loose heads, abnormal contours, abnormal midpieces, nuclear vacuoles, double forms and dag defects) and minor defects (small normal heads, normal loose heads, abaxial implantation, coiled tails and distal droplets). A total of 200 cells (%, DIC microscopy, oil immersion, 1000×, Olympus BX61), recording one abnormality per cell, were examined per sample.

#### Slow thermoresistance test (STT)

After thawing, the samples were transferred to a glass tube, and motility, vigour and DNA integrity were assessed. Subsequently, the semen was incubated for 3 hours at 37°C; after incubation, motility, vigour and DNA integrity were assessed again, as was the percentage of intact acrosomes (PIA) (%, DIC microscopy, oil immersion, 1000×, Olympus BX61) [13].

### DNA integrity

Chromatin integrity was assessed using the acridine orange (AO) technique described by Tejada et al. [14] and modified by Unanian [15], who washed the samples with distilled water. Acridine orange is a DNA metachromatic stain that permits the detection of DNA fragmentation by colour differences (green for intact chromatin and yellow to red for damaged DNA).

The staining solution was prepared from a 10-ml stock of AO (1 g of AO, Sigma, St. Louis, MO, USA, diluted in 1000 ml of distilled water, and stored at 4°C in the dark), 40 ml of 0.1 M citric acid anhydrous solution, 2.5 ml of 0.3M Na_2_HPO_4 _solution and pH 2.5.

Semen samples (100 μl) were centrifuged with 100 μl of distilled water at 300 g for 5 minutes to remove the egg-yolk extender and to promote damage to the plasma membranes. Semen pellets were washed twice, and two slides (one was stored for security) were prepared and dried at room temperature for 60 min. Dried smears were submersed in Carnoy's solution (three parts methanol to one part acetic acid). After 12 h of fixation, the slides were stained for five minutes with 3 ml of acridine orange solution (10 ml acridine orange, 1 μg/ml; 40 ml citric acid 0.1M; 2.5 ml sodium phosphate 0.3M, pH 2.5) and protected from light exposure. The slides were analysed under an epifluorescence microscope (Olympus BX61 - Tokyo, Japan - excitation 460-570 nm and emission 460-610 nm; 400×). For each smear, 400 cells were classified as follows: cells emitting green fluorescence were considered to have intact chromatin, and cells emitting red, orange or red/orange fluorescence, distributed in a regular or irregular pattern within the sperm head, were classified as spermatozoa with partial or total chromatin denaturation [16]. A DNA integrity assay was repeated at the end of the STT (37°C/3 h).

### Plasma membrane integrity, acrosome integrity and mitochondrial potential

Plasma membrane, acrosomal integrity and mitochondrial function were evaluated using the techniques described by Celeghini et al. [17] and Gonçalves et al. [18], which involve the combination of propidium iodide (PI, Sigma, St. Louis, MO, USA), fluorescein-conjugated *Pissum sativum *agglutinin (FITC-PSA, Sigma, MO, USA) and JC-1 (Sigma, St. Louis, MO, USA).

The solutions were prepared according to Celeghini et al. [17]; briefly, a 25 mg/ml PI stock solution was prepared in dimethyl sulphoxide (DMSO) and stored at -20°C, the working solution was diluted to 0.2 mg PI/ml Dulbecco's phosphate buffer (DPBS) (-20°C, in the dark).

The FITC-PSA working solution was prepared adding 100 μg FITC-PSA to 1 ml of DPBS with 10% sodium azide solution (10%) and kept at 4°C in the dark. For the JC-1 stock solution 5 mg of JC-1 was mixed to 1 ml of DMSO; for staining, 200 μl of stock solution was added to 800 μl of DMSO in a final concentration of 0.1 mg/ml (153 μM) of JC1 and stored at -20°C in the dark.

Thawed semen samples (250 μL) were transferred to 500 μl of modified Tyrode's albumin lactate pyruvate (TALP; [19]) and centrifuged at 300 g for 5 minutes. The sediment was resuspended and centrifuged twice in 250 μl of TALP. A 30-μl aliquot of diluted semen in TALP medium (25 × 10^6^sptz/ml) was transferred to a warmed microtube containing 2 μl of PI (0.2 mg/ml), 1.6 μl of JC-1 (0.5 mg/ml) and 10 μl of FITC-PSA (100 μg/ml). The samples were incubated at 37°C for eight minutes and protected from light exposure. An 8-μl sample of the stained spermatozoa suspension was placed on a slide, coverslipped and immediately analysed under an epifluorescence microscope (Olympus BX61 - Tokyo, Japan, 400×) with an excitation filter at 460-570 nm and an emission filter at 460-610 nm. Two hundred cells were examined and classified based on the fluorescence emitted from each probe. Propidium iodide-positive cells (damaged plasma membrane) showed a red-stained nucleus, FITC-PSA-positive cells (damaged acrosome) presented a yellow-green acrosome region, JC-1 -positive cells (high mitochondrial potential) showed a bright red-orange colour in the mid-piece region, and JC-1-negative cells (low mitochondrial potential) presented a bright green colour in the mid-piece region.

### Oocyte collection and maturation

Bovine ovaries were obtained in a local abattoir and transported to the laboratory. Intact cumulus-oocyte complexes were aspirated from antral follicles (2-8 mm in diameter). Oocytes with at least four layers of cumulus cells were selected for the experiments. Oocytes (20 oocytes per droplet) were washed and cultured in 100-μl droplets of *in vitro *maturation (IVM) medium, which consisted of TCM-199 (Gibco BRL, NY, USA) supplemented with 10% (v/v) foetal calf serum (FCS; Gibco BRL, NY, USA), 0.2 mM sodium pyruvate, 25 mM sodium bicarbonate, 50 μg/ml amikacin, 0.5 μg/ml FSH (Pluset^®^; HertapeCalier, MG, Brazil), 100 IU/ml hCG (Profasi^®^; Serono, SP, Brazil) and 1.0 μg/ml estradiol-17β. Culture was carried out for 24 h at 38.5°C under an atmosphere of 5% CO_2_, in air with maximum humidity [20-22].

### Sperm preparation and in vitro fertilisation (IVF)

Five bulls were selected for IVF: three from group 1, all of them showing 0% PCDs in semen, and two from group 2, with 28.5% and 40.5% spermatozoa with PCDs. The IVF procedure was repeated six times, and the semen of all five bulls was used in all of the iterations. For IVF, frozen-thawed semen was prepared by washing twice at 300 g for 5 min in pre-warmed TALP at 38.5°C. The washed semen was added to the fertilisation drops at a final concentration of 2 × 10^6 ^sperm cells/ml of IVF medium, which consisted of TALP supplemented with 0.2 mM Na-pyruvate, 6 mg/ml fatty acid-free bovine serum albumin (BSA), 25 mM sodium bicarbonate, 13 mM Na-lactate, 50 μg/ml amikacin, 4 μl/ml PHE solution (1 mM hypotaurine, 2 mM penicillamine and 250 μM epinephrine) and 10 μg heparin. Oocytes and sperm were co-incubated at a concentration of 5 × 10^3 ^sperm cells/oocyte (20 oocytes per 100-μl droplet and 100 × 10^3 ^sperm cells/4 μl medium) for 18 h under the same temperature and atmospheric conditions used for IVM [20-22].

### Embryo culture

Following fertilisation, the presumptive zygotes were transferred to *in vitro *culture medium (IVC) consisting of modified oviductal fluid [23] supplemented with 50 μg/ml amikacin, 5 mg/ml fraction fatty acid-free BSA and 2.5% FCS. Zygotes were incubated under mineral oil up to 48 h post-insemination (hpi) for the assessment of cleavage rates under stereoscopic microscopy (40×), when two- and four-cell embryos were counted. Blastocyst development rates were recorded at day 7 (168 hpi), and hatched blastocysts were counted at day 8 (192 hpi) IVC. The temperature and gas atmosphere were the same as used for IVM and IVF [20-22].

## Statistical analyses

The null hypothesis (H_0_) was that there were no differences between bulls with high and low levels of PCD regarding sperm quality traits and embryo production efficiency. Statistical analyses were performed using the Statistical Analysis System software (release 9.2. SAS Institute Inc., Cary, NC, USA, 2008). Data (except vigour) were transformed in arcsin$\sqrt{\%}$ to obtain a normal distribution. Concentration, PIA, membrane integrity, acrosome integrity, mitochondrial potential and IVF results were examined using two-way ANOVA (bull × group); for the STT, results (motility, vigour and DNA integrity) were tested by MANOVA (bull × group × incubation time). The means were compared using least square methods. The correlation coefficient (r) and determination factor (r^2^) were calculated for motility, vigour, membrane and acrosome integrity, mitochondrial potential and DNA integrity. The data were presented as means ± SDs and were considered statistically significant when P < 0.05 [24].

## Results

The post-thaw semen quality of the five bulls from group 1 and eight bulls of group 2 are summarised in Tables 1 and 2. No statistical differences were seen between the groups for sperm concentration and the percentage of minor morphological defects; nevertheless, as expected, total major defects and the percentage of PCDs were higher for group 2 - PCD (P < 0.05) (Table 1). The total morphological defects percentages can be mainly attributed to the higher PCD levels as seen by the determination factor (0.93) and the regression equation (total morphological defects = 11.69 +1.41PCD).

**Table 1:** Summary of semen traits (mean ± SD) for group 1 (n = 5) and group 2 (n = 8)


	**Group 1 (Control)**	**Group 2 (PCD)**

**Sperm concentration (million/ml)**	29.0 ± 4.1^a^	31.8 ± 8.0^a^
**Major sperm defects (%)**	4.7 ± 2.6^a^	38.6 ± 10.8^b^
**Minor sperm defects (%)**	5.4 ± 2.2^a^	9.2 ± 6.1^a^
**Total sperm defects (%)**	10.1 ± 3.4^a^	47.9 ± 13.3^b^
**PCD (%)**	0.5 ± 0.6^a^	24.6 ± 11.3^b^

Means with different superscripts in the same line differ significantly (P < 0.05).

**Table 2:** Mean ± SD percentage of progressively motile spermatozoa, sperm vigour, percentage of spermatozoa with intact acrosomes and DNA damage in semen samples with low (group 1, control) and increased percentages of proximal cytoplasmic droplets (PCD, group 2) before (0h) and after 3h of incubation at 37°C


	**Motility (%)**		**Vigour (0-5)**		**PIA (%)**	**DNA damage (%)**	
	
	**0h**	**3h**	**0h**	**3h**	**3h**	**0h**	**3h**

**Group 1 (n = 5)**	45.3 ± 6.7^a^	35.0 ± 8.2^b^	4.5 ± 0.1ª	3.8 ± 0.7^b^	54.6 ± 6.9	0,5 ± 0,3	1,0 ± 0,4
**Group 2 (n = 8)**	40.8 ± 17.1^a^	25.4 ± 13.3^b^	4.0 ± 1.3^a^	3.1 ± 1.4^b^	42.7 ± 17.5	0,7 ± 0,4	1,4 ± 1,3

Means with different superscripts in the same line differ significantly (P < 0.05).

The percentage of progressively motile spermatozoa and vigour before and after STT, as well as the percentage of spermatozoa with intact acrosomes analysed by PIA method (PIA), were not different between groups 1 and 2. However, when the correlation coefficient and determination factors were calculated for these traits, significant values (P < 0.01) were found (Table 3).

**Table 3:** Regression, correlation coefficients and levels of significance for proximal cytoplasmic droplets (PCD) versus sperm quality traits


	**Y = a + bPCD**				
	
	**a**	**b**	**r**^**2**^	**r**	**P**

**Motility 0h**	49.65	-0.47	0.24	-0.50	0.0012
**Motility 3h**	36.71	-0.50	0.35	-0.59	< 0.0001
**Vigour 0h**	4.83	-0.04	0.33	-0.58	< 0.0001
**Vigour 3h**	4.11	-0.05	0.34	-0.58	< 0.0001
**PIA 3h**	58.15	-0.71	0.46	-0.68	< 0.0001
**Damaged DNA 0h**	0.47	0.02	0.16	0.40	0.0103
**Damaged DNA 3h**	0.28	0.05	0.26	0.51	0.0006
**IIH**	27.71	-0.37	0.59	-0.77	< 0.0001
**DDL**	51.60	0.44	0.30	0.54	0.0002
**Damaged acrosome**	62.41	0.27	0.22	0.47	0.0020
**Damaged sperm membrane**	64.58	0.47	0.48	0.69	< 0.0001
**Low mitochondrial function**	61.30	0.54	0.48	0.70	< 0.0001

PIA 3 h - Percentage of intact acrosomes after 3h of incubation at 37°C

Damaged DNA 0h -- before incubation

Damaged DNA 3h -- after 3h of incubation at 37°C

IIH -- Intact acrosome, intact membrane and high mitochondrial potential

DDL -- Damaged acrosome, damaged membrane and low mitochondrial potential

The semen samples from group 2 (PCD) showed higher levels (P < 0.05) of spermatozoa with simultaneously damaged acrosomes, damaged membranes and low mitochondrial potential; furthermore, group 1 (control) had a higher percentage (P < 0.05) of cells with positive characteristics, such as intact membranes, intact acrosomes and high mitochondrial potential (Table 4). With regard to damaged acrosomes, plasma membranes and low mitochondrial potential, the PCD group presented higher values than group 1 (P < 0.05).

**Table 4:** Mean ± SD percentage of spermatozoa with simultaneous intact acrosome, intact membrane and high mitochondrial potential (IIH) or damaged acrosome, damaged membrane and low mitochondrial potential (DDL) and summarised data for damaged acrosome and sperm membrane and low mitochondrial function in semen samples of group 1 (Control) and group 2 (PCD)


**Spermatozoa with**	**Group 1 (n = 5)**	**Group 2 (n = 8)**

IIH (%)	28.9 ± 3.1	17.7 ± 5.3*
DDL (%)	48.4 ± 8.4	64.5 ± 9.2*
Damaged acrosome (%)	61.5 ± 3.2	70.3 ± 7.8*
Damaged sperm membrane (%)	61.8 ± 3.2	78.5 ± 5.3*
Low mitochondrial function (%)	58.1 ± 3.8	76.7 ± 5.4*

*P < 0.05

IIH -- Intact acrosome, intact membrane and high mitochondrial potential

DDL -- Damaged acrosome, damaged membrane and low mitochondrial potential

No significant differences were found between group 1 and group 2 regarding sperm chromatin status before and after STT (Table 2). Nevertheless, the correlation between PCDs and DNA damage was significant and was even higher after STT (Table 3). Regarding *in vitro *fertilisation results for semen samples from group 1 (n = 3) and group 2 (n = 2), the rates of cleavage, blastocyst formation and hatched blastocysts showed no significant differences (Table 5). However, the percentages of hatched blastocysts differed between bulls C1, C3 and P1 (18.0% ± 79.7, 16.5% ± 7.9, and 16.1% ± 11.1, respectively) at one extreme and bulls C2 and P2 (2.5% ± 3.6 and 2.7% ± 3.5, respectively) at the other (Table 6).

**Table 5:** Mean ± SD rates of cleavage, blastocyst formation (BL) and hatched blastocyst per cleavage rate (BLH/CV) for group 1 (control) and group 2 (spermatozoa with increased percentages of cytoplasmic droplets - PCD)


	**Oocyte (n)**	**Cleavage (%)**	**Blastocyst (%)**	**Hatched BL (HBL/CV-%)**

**Group 1**	558	63.6 ± 15.4	33.4 ± 15.7	13.4 ± 10.0
**Group 2**	505	60.3 ± 22.2	29.8 ± 14.2	8.9 ± 10.3

(P > 0.05)

**Table 6:** Mean ± SD percentage of spermatozoa with proximal cytoplasmic droplets (PCDs), number of oocytes, cleavage, blastocyst formation (BL) and hatched blastocyst per cleavage rate (HBL/CV) for bulls C1, C2 and C3 from group 1 (control) and for bulls P1 and P2 with increased PCD (group 2)


**Bull**	**PCD (%)**	**Oocyte (n)**	**Cleavage (%)**	**Blastocyst (%)**	**Hatch BL (HBL/CV-%)**

**C1**	0.0	201	71.5 ± 13.8 ª	37.4 ± 15.2 ª	18.0 ± 9.7 ^a^
**C2**	0.0	138	57.0 ± 18.2 ^ab^	19.8 ± 8.2 ^b^	2.5 ± 3.6 ^b^
**C3**	0.0	219	59.4 ± 13.5 ^ab^	39.5 ± 16.1 ª	16.5 ± 7.9 ^a^
**P1**	28.5	210	72.0 ± 17.3 ª	39.6 ± 11.3 ª	16.1 ± 11.1 ^a^
**P2**	40.5	295	50.2 ± 21.8 ^b^	21.3 ± 10.8 ^b^	2.7 ± 3.5 ^b^

Means with different superscripts in the same column differ significantly (P < 0.05).

## Discussion

Previous studies have related spermatozoa with increased percentages of PCDs to lower motility in humans [25]. A similar connection was found in the present study, as seen by the significant (P < 0.001) negative correlation between motility and PCDs. In young beef bulls, zero to six weeks after puberty, the percentage of progressively motile spermatozoa increased rapidly, while the percentage of spermatozoa with proximal cytoplasmic droplets was correspondingly dramatically reduced [26]. High percentages of ejaculated spermatozoa with retained cytoplasmic droplets are associated with infertility in adult bulls [3,8,9]. A recent review indicated that elimination of the droplets during ejaculation may be prognostic for fertility, while their retention may indicate sub- or infertility [27].

Morphologically abnormal spermatozoa are more susceptible to oxidative stress. The production of reactive oxygen species (ROS) by spermatozoa with PCDs can induce oxidative stress [28], resulting in cellular dysfunction by a number of mechanisms, among them membrane lipid peroxidation [1]. Lipidic peroxidation was observed *in situ *in human [29] and bovine [28] spermatozoa midpieces, incorporating the mitochondria and any retained excess residual cytoplasm [29,30]. The impact of the cryopreservation process on membranes and mitochondria has been intensely studied [31-36]. However, there are few studies indicating that semen with specific morphological alterations would be more sensitive to injuries on these compartments [7,29,36].

Considering these data, the high percentages of spermatozoa with damaged plasma membranes and acrosomes and with low mitochondrial potential, as observed in group 2 for semen samples with high proportions of PCDs, may have been due to higher production of ROS, resulting in oxidative damage. Although differences were observed in the results obtained for acrosome integrity as analysed using the PIA and triple staining techniques, this discrepancy was due to different methodologies; PIA evaluates the percentage of detached acrosomes after STT (37°C/3h) [13], while the FITC-PSA fluorescent probe binds the glycoconjugates and thus labels the acrosome content and indirectly determines acrosome integrity [37]. Nevertheless, the correlation in the results indicates that PCDs have an important effect on acrosome integrity, regardless of the evaluation method.

Semen DNA integrity did not differ between group 1 and group 2; however, the results indicated a higher correlation between the percentage of spermatozoa with PCDs and stable DNA, in particular after STT, indicating that chromatin susceptibility to acid denaturation may be greater after incubation at 37°C, as observed in our previous study [38].

Considering the discrepancies among IVF results, individual responses in the IVF process can be assumed. In fact, the bull effect in this experiment was not prevented by pooling the semen. Grouping the bulls according to percentages of spermatozoa with PCDs (group 1/control and group 2/high levels of PCD), the rates of cleavage, blastocyst formation and hatched blastocysts did not significantly differ. A high incidence of proximal droplets is indicative of impaired epididymal maturation in young bulls and may severely compromise IVF results because when they reach sexual maturity, proximal droplets decrease and IVF potential increases [3]. However, the bulls used in the present study were adults and were sexually mature.

Considering the results from IVF of the two bulls with higher PCD levels (bulls P1 = 28.5% PCD and P2 = 40.5% PCD), P2 had a numerically lower performance in IVF, indicating that the higher levels of PCD may have affected the embryo production efficiency of this animal (P2). Thundathil et al. [7] suggested that morphologically normal sperm co-existing in the semen along with spermatozoa showing proximal droplets were also functionally deficient. These authors used semen with even higher levels of PCDs (45% to 86%) and found low cleavage rates (8% to 15%) and no blastocyst formation. Nevertheless, the results for control bull C2 were similar to those for the high PCD bull, P2, and this result indicates that, despite our hypothesis regarding the influence of PCDs on IVF rates, the results may have been affected by different variables, such as individual bull characteristics. Bull effects on sperm motility [39], IVF results and embryo development rates have been observed in a number of investigations [19,40-44]. Both fertilising ability and competency for embryonic development have varied significantly among the semen of individual bulls [41]. Individual semen characteristics were found not to affect cleavage but to affect embryo development [40].

Our results emphasise the importance of the genetic background of individual semen donors for successful IVF procedures, as well as careful semen evaluation.

## Conclusions

This study indicated that higher levels of PCDs could influence the sperm quality traits; nevertheless, this effect was not sufficient to impair motility and vigour, but it did affect the integrity of the acrosomal membrane and plasma membrane and resulted in low mitochondrial potential after thawing. Chromatin was not affected overall by higher levels of PCDs, but it showed a greater correlation with damage, especially after 3 h of incubation at 37°C. As previously stated, IVF and embryo production results may have been affected by the interaction of morphology traits and individual bull effects.

## Competing interests

The authors declare that they have no competing interests.

## Authors' contributions

JTC carried out the semen evaluation and IVF procedures and wrote the manuscript; GZM coordinated the IVF procedure; LHR and CR participated in semen collection, freezing and evaluation; SHVP performed the statistical analyses; and MBK was the organiser and the principal investigator. All the authors have read and approved the final manuscript.
